# Evidence for in vivo resistance against allopurinol in a dog infected with *Leishmania infantum* by reduction in copy numbers of the *S*-adenosylmethionine synthetase (*METK*) gene

**DOI:** 10.1186/s13071-024-06583-0

**Published:** 2024-12-16

**Authors:** Ingo Schäfer, Mathieu Faucher, Yaarit Nachum-Biala, Lluís Ferrer, Marina Carrasco, Alexandra Kehl, Elisabeth Müller, Torsten J. Naucke, Gad Baneth

**Affiliations:** 1 LABOKLIN GmbH and Co. KG, Bad Kissingen, Germany; 2 Clinique Veterinaire Alliance, Bordeaux, France; 3 Koret School of Veterinary Medicine, The Hebrew University, Rehovot, Israel; 4 NANO1HEALTH SL, Parc de Recerca UAB, 08193 Bellaterra, Barcelona Spain

**Keywords:** Vector-borne infection, Leishmaniasis, Treatment

## Abstract

**Background:**

In Europe, canine leishmaniasis is commonly caused by Leishmania infantum. Allopurinol is the main drug for long-term management of the disease, and clinical relapses of *L. infantum* infection treated with this drug are described. Resistance to allopurinol has been demonstrated in-vitro, but there is only little knowledge on in vivo resistance in dogs.

**Findings:**

A two-year-old female spayed Akita Inu that was adopted from a breeding facility near Nice in France was initially diagnosed with primary immune-mediated hemolytic anemia. Immunosuppressive treatment was initiated, and the dog was referred for a second opinion to the Clinique Veterinaire Alliance in France. PCR testing for *L. infantum* was performed out of EDTA blood and IFA as well as ELISA testing out of serum. Resistance to allopurinol was associated with chromosome and gene copy number (CN) variations including a decrease in the S-adenosylmethionine synthetase (*METK*) gene CN.

**Results:**

The dog showed pale mucous membranes, fever (39.1 °C), and a relapse of the anemia. The diagnosis of leishmaniasis was based on the cytological finding of *Leishmania* amastigotes (bone marrow, spleen, liver), positive PCR testing, and positive IFAT serology. The dog was treated with allopurinol over a period of 1316 days and additionally received two cycles of Glucantime® (meglumine antimoniate), before samples were submitted to the LABOKLIN laboratory to test for resistance against allopurinol. The laboratory work-up revealed mild thrombocytopenia, mild hyperproteinemia with hyperglobulinemia, a marked elevation of the c-reactive protein, and decreased iron concentration. Serum protein electrophoresis showed a polyclonal peak in the gamma globulins. Serology was positive in both ELISA (21.5 LE) and IFAT (1:1024). Quantitative PCR testing of blood was positive with low numbers of Leishmania (10/ml blood) at the timepoint of suspicion for resistance. The urinary protein-to-creatinine ratio was markedly elevated (2.5) and xanthine crystalluria was detected. A CN level of below 3 is considered suspicious for resistance, as revealed in the described Akita Inu dog.

**Conclusions:**

Relapse of *L. infantum* infection after applying allopurinol for 1316 days due to resistance was suspected clinically. Positive PCR testing, consistent hematological and biochemistry abnormalities, and reduction in the *METK* gene CN backed up the clinical suspicion of resistance. Dogs infected with allopurinol resistant strains of *L. infantum* may represent a great risk for infection of naïve dogs, cats, and humans.

**Graphical Abstract:**

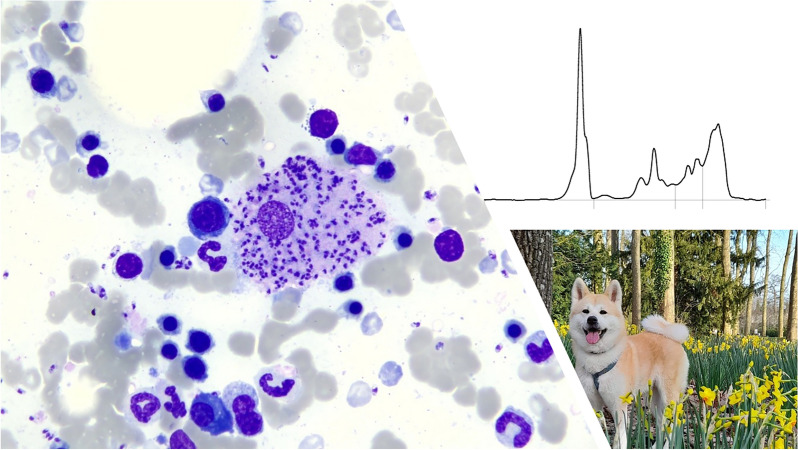

## Main text

### Introduction

Leishmaniasis is a protozoal disease caused by *Leishmania infantum* in humans, dogs, and cats in Europe [1–4]. In general, leishmaniasis is considered a globally emerging disease, with phlebotomine sandflies as primary vectors and dogs as the main pathogen reservoir [1, 2]. In addition to vector transmission, blood transfusion [5–9], vertical transmission [10], and venereal transmission [11] have been proven as sources for canine *L. infantum* infections. The purine analog allopurinol acts as a xanthine oxidase inhibitor to reduce the serum urate concentration, and is used for the management of gout in human medicine [12]. In canine leishmaniasis, antileishmanial activity of allopurinol was shown by inhibition of the leishmanial enzyme hypoxanthine–guanine phosphoribosyl transferase, leading to a disruption in protein translation and selective death of leishmanial parasites [13, 14]. In veterinary medicine, allopurinol is the first-line drug of choice for long-term management of canine leishmaniasis, applied alone or in combination with pentavalent antimonials or miltefosine [15, 16]. While allopurinol is rarely used in human medicine for the treatment of human leishmaniasis, it is the only drug recommended for use in dogs by the World Health Organization (WHO) [1]. In several previous studies, no direct association between clinical disease relapse in dogs with leishmaniasis and drug resistance was demonstrated [17–19]. However, in 2016, a first detailed report of resistance to allopurinol in *L. infantum* parasites isolated from dogs and associated with clinical relapse of disease was published [20]. Resistance was demonstrated in three forms of *L. infantum* parasites [20]. Allopurinol resistance has been successfully induced in vitro in *L. infantum* isolates from dogs cultured under increasing drug pressure, and genetic modifications were identified in resistant isolates [21, 22].

### Methods

TaqMan^®^ real-time PCR testing for detection of *L. infantum* was performed at LABOKLIN (Bad Kissingen, Germany) using a LightCycler 96 (Roche Diagnostics, Mannheim, Germany). Cycle threshold (Cq) values below 35 were considered positive. Each PCR run included a negative and a positive control, as well as an extraction control in each sample, to check for nucleic acid extraction and PCR inhibition (DNA/RNA Process Control Detection Kit, Roche Diagnostics GmbH, Mannheim, Germany). EDTA-blood was used for PCR testing (target: kinetoplast minicircle DNA; primer: 5′-AAC TTT TCT GGT CCT CCG GGT AG-3′, 5′-ACC CCC AGT TTC CCG CC-3′; probe: 5′-FAM-AAA AAT GGG TGC AGA AAT-NFQMGB-3′ [21]). For serological detection of *Leishmania* spp., enzyme-linked immunosorbent assay (ELISA) testing (NovaTec VetLine Leishmania ELISA, Immundiagnostica GmbH, Dietzenbach, Germany, > 11 LE positive) and indirect fluorescent antibody (IFA) testing (MegaFLUO^®^ LEISH, MegaCor Diagnostik GmbH, Hörbranz, Austria; > 1:64 positive) were used on serum.

Primers and probes specific for the *METK* and glyceraldehyde-3-phosphate dehydrogenase (*GAPDH*, as reference gene) genes of *L. infantum* were designed and tested in ddPCR using Supermix, DropletGenerator, and Droplet Reader (BioRad, Feldkirchen, Germany) in the LABOKLIN laboratory (Bad Kissingen, Germany). Cross-reactivity with canine DNA could be ruled out by the absence of amplification of the *METK* gene in samples from dogs that tested negative for *Leishmania*. Accuracy was tested and confirmed by quantifying the copy number of *METK* in the WHO strain MHOM/TN/80/IPT-1, described as having only one CN of *METK* [22]. Samples were sent to NANO1HEALTH SL (Barcelona, Spain) for additional investigation of CNs of the *METK* gene.

Additionally, a complete blood count (Sysmex XN-V analyzer, Sysmex Deutschland, Norderstedt, Germany), a biochemical profile including CRP (Cobas 8000, Roche Deutschland Holding GmbH, Mannheim, Germany), and a serum protein capillary electrophoresis (Sebia Minicap, Sebia, Mainz, Germany) were performed in the LABOKLIN laboratory. Urinalysis was done on the Cobas u601 (Roche Deutschland Holding GmbH, Mannheim, Germany), with an additional microscopical examination of the urinary sediment and a urinary protein-to-creatinine ratio (Cobas 8000, Roche Deutschland Holding GmbH, Mannheim, Germany).

### Case report

**Fig. 1:**
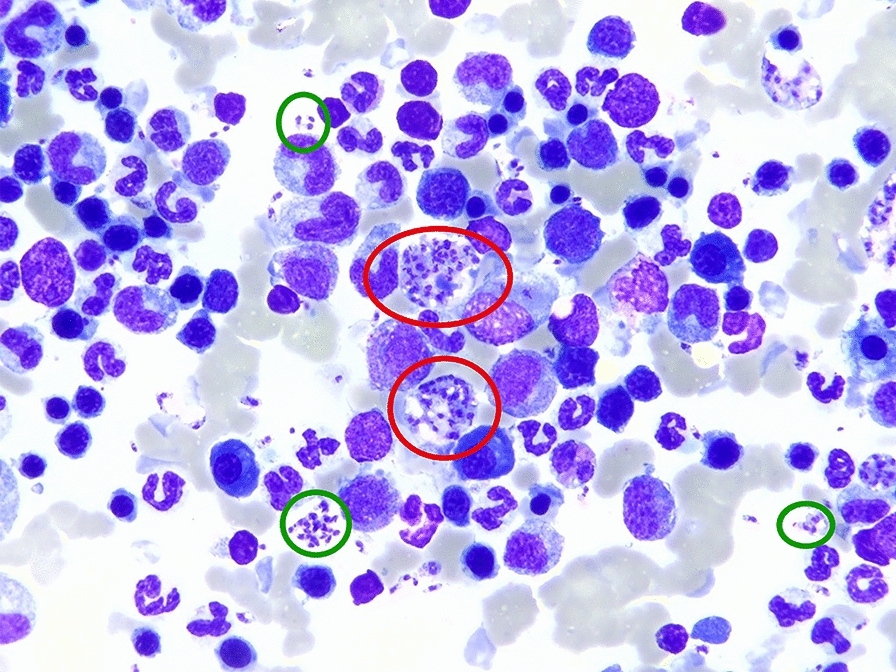
Cytology of the bone marrow of a 2-year-old female, spayed Akita Inu infected with *Leishmania infantum* with numerous intracellular (red circles) and extracellular amastigotes (green circles)

A 2-year-old female, spayed Akita Inu dog was adopted from a breeding facility near Nice located at the southeastern coast of France on the Mediterranean Sea. This part of France is endemic for *L. infantum*, with the highest reported incidences in humans and dogs in the country [23]. The dog was referred for a second opinion to the veterinary clinic Clinique Veterinaire Alliance in Bordeaux, France, with a diagnosis of a primary immune-mediated hemolytic anemia (IMHA). The dog had been treated for IMHA for 5 months, receiving cyclosporine 10 mg/kg once daily orally and prednisolone 0.8 mg/kg once daily orally, when tested for leishmaniasis (Table 1). At the time IMHA was diagnosed, a screening for additional vector-borne pathogens was carried out without revealing coinfections (PCR out of EDTA blood: *Babesia* spp., *Borrelia* spp., *Ehrlichia canis*, *Anaplasma phagocytophilum*, *A. platys*; SNAP 4Dx test [IDEXX laboratories, Inc., Maine, USA]: antibodies for *Borrelia burgdorferi*, *A. phagocytophilum*, *A. platys*, *E. canis*; and antigen testing for *Dirofilaria immitis*). The owners had decreased the dosage of the immunosuppressive treatment, and a relapse of the anemia occurred. Physical examination revealed pale mucous membranes and a body rectal temperature of 39.1 °C, plus a mild nonregenerative anemia (hematocrit 0.27 l/l, reference range [RR] 0.37–0.63 l/l), thrombocytopenia (121 × 10^9^/l, RR 148–484 × 10^9^/l), mild hyperproteinemia (87 g/l, RR 52–82 g/l) with hyperglobulinemia (52 g/l, RR 25–45 g/l), and albumin in the RR (25 g/l, RR 23–40 g/l, Table 2). A urinary protein-to-creatinine ratio (UPC) of 0.66 (RR < 0.3) was noted. Thoracic imaging was unremarkable, and a mottled parenchyma of the spleen, hyperechoic hepatomegaly, and small adrenal glands were noted on abdominal ultrasound. Fine needle aspirate cytology showed lymphoid hyperplasia, granulomatous inflammation, and numerous *Leishmania* sp. amastigotes in the spleen as well as in the bone marrow (Fig. 1) and, to a smaller extent, also in the liver. Additionally, the dog tested serologically positive for *L. infantum* by IFAT (1:640) in the Orbio laboratory (Bron, France) (Tables 1, 2), and treatment was started with the antileishmanial drugs allopurinol and meglumine antimonate.

**Table 1:** Course of treatment in a 2-year-old female, spayed Akita Inu infected with *Leishmania infantum*

Day	Treatment	Diagnosis	*L. infantum* status
−5 months	Cyclosporine 10 mg/kg once daily orally Prednisolone 0.8 mg/kg once daily orally	Primary immune-mediated hemolytic anemia	—
Day 0 (first presentation)	Allopurinol 9.3 mg/kg twice daily orally Meglumine antimonate 92 mg/kg once daily subcutaneously^a^ Prednisolone 0.6 mg/kg once daily orally Stopped cyclosporine	Leishmaniasis	Serology 1:640 (positive)^b^
Day 38	Allopurinol 9.3 mg/kg twice daily orally Meglumine antimonate 92 mg/kg once daily subcutaneously Prednisolone 0.4 mg/kg once daily orally		—
Day 73	Allopurinol 7.4 mg/kg twice daily orally Prednisolone 0.2 mg/kg once daily orally Meglumine antimonate finished		—
Day 143	Allopurinol 7.4 mg/kg twice daily orally Prednisolone stopped		Serology 1:640 (positive)^b^
Day 679	Allopurinol 7.4 mg/kg twice daily orally Meglumine antimonate 80 mg/kg once daily subcutaneously		Serology 1:640 (positive)^b^ PCR positive^b^
Day 722	Allopurinol 7.4 mg/kg twice daily orally Meglumine antimonate finished		—

^a^Glucantime 1.5 g/5 ml, Sanofi-Aventis France, Gentilly Cedex

^b^Orbio laboratory, Bron, France

**Table 2:** Body weight, complete blood count, biochemical parameters, and parasitological status in a 2-year-old female, spayed Akita Inu infected with *Leishmania infantum* from first presentation (day 0, D0) to day 1316 (D1316) recorded in the Clinique Veterinaire Alliance (Bordeaux, France)

Parameter	RI	D0	D38	D73	D143	D244	D351	D416	D625	D679	D736	D875	D1050	D1270	D1316
BW (kg)	—	27.0	27.4	—	26.0	24.3	25.2	24.4	—	22.9	23.6	25.5	26.0	25.3	26.2
Change in BW (%)	—	100.0	101.5	—	96.3	90.0	93.3	90.4	—	84.8	87.4	94.4	96.3	93.7	97.0
Complete blood count^a^															
RBC (× 10^12^/l)	5.65–8.87	**5.08**	6.93	7.35	7.14	8.12	8.31	—	7.22	—	7.29	7.53	8.3	—	8.29
HGB (g/l)	131–205	**97**	**128**	143	138	160	156	—	**125**	—	**113**	141	154	—	**125**
HCT (l/l)	0.37–0.62	**0.27**	0.37	0.39	0.39	0.46	0.46	—	0.37		**0.35**	0.42	0.47	—	0.38
WBC (× 10^12^/l)	5.1–16.8	12.9	9.7	7.0	6.7	6.4	7.1	—	8.9	—	**16.9**	6.1	**4.14**	—	6.1
Seg (× 10^9^/l)	3.0–11.6	10.2	7.8	4.0	4.3	3.2	3.8	—	5.8	—	**12.9**	**2.9**	**1.9**	—	3.4
Lymphs (× 10^9^/l)	1.1–5.1	1.8	1.5	2.4	2.0	2.7	2.9	—	2.6	—	2.8	2.5	1.9	—	2.4
Eos (× 10^9^/l)	0.06–1.23	**0.01**	**0.01**	0.20	0.15	0.24	0.19	—	0.14	—	0.25	0.41	0.19	—	0.09
Monos (× 10^9^/l)	0.2–1.1	0.8	0.4	0.3	0.3	0.2	0.2	—	0.3	—	0.9	0.2	**0.1**	—	0.2
CHr (pg)	22.3–29.6	**17.0**	**17.8**	**18.6**	**18.0**	**17.7**	**21.4**	—	**17.4**	—	**20.6**	**21.9**	**21.9**	—	**17.0**
THR (× 10^9^/l)	148–484	**121**	201	166	241	**144**	**125**	—	**119**	—	165	**142**	**107**	—	**74**
Ret (× 10^9^ /l)	10.0–110.0	63.5	83.9	17.6	75.7	78.0	46.5	—	69.3	—	44.5	29.2	67.2	—	38.1
Biochemistry^b^															
TP (g/l)	52–82	**87**	**83**	72	—	72	70	—	81	**88**	82	69	73	84	**87**
Alb (g/l)	23–40	25	40	36	—	33	32	—	30	28	29	32	34	28	29
Glob (g/l)	25–45	**52**	43	36	—	39	38	—	**51**	**60**	**53**	37	39	**56**	**58**
A/G	—	0.7	0.9	1.0	—	0.8	0.8	—	0.6	0.5	0.5	0.9	0.9	0.5	0.5
Urea (g/l)	0.15–0.57	0.32	—	—	—	—	—	—	0.18	—	—	—	—	0.09	—
Crea (mg/l)	5.0–18.0	8.7	6.0	10.2	12.0	15.0	**18.1**	15.2	14.4	**23.3**	**19.4**	16.7	16.5	15.0	**18.7**
Bil (mg/l)	< 9.0	3.5	—	—	—	—	—	—	1.6	—	—	—	—	1.0	—

Bold indicates values outside reference intervals

*A/G* albumin-to-globulin ratio, *Alb* albumin, *Bil* bilirubin, *BW* body weight, *CHr* reticulocyte hemoglobin content, *Crea* creatinine, *Eos* eosinophilic granulocytes, *Glob* globulin, *HCT* hematocrit, *HGB* hemoglobin, *Lymphs* lymphocytes, *Monos* monocytes, *RBCs* red blood cells, *Seg* segmented neutrophilic granulocytes, *Ret* reticulocytes, *THR* platelets, *TP* total protein, *WBCs* white blood cells

^a^IDEXX ProCyte One Hematology Analyzer

^b^IDEXX Catalyst One Chemistry Analyzer

The dog’s course of treatment is shown in Table 1, and the results of the bloodwork in Table 2. The immunosuppressive therapy with cyclosporine was stopped as soon as leishmaniasis was diagnosed, while prednisolone dosage was tapered over 2 months before it was stopped as well (Table 1). Physical examination of the dog on day 38, after the first presentation in the veterinary clinic (Clinique Veterinaire Alliance), was unremarkable, indicating clinical improvement. On day 73, the urine sediment revealed a xanthine crystalluria in urinalysis, and a low purine diet was applied (Urinary U/C low purine, Royal Canin, Aimargues, France). Meglumine antimonate treatment was finished, and the dog continued to be managed on allopurinol (Table 1). On day 351 after the first presentation, the owner did not report any clinical abnormalities; however, mild azotemia (18.1 mg/l; RR 5.0–18.0) with a urinary specific gravity of 1.016 (RR > 1.025), a mild leukocyturia, and xanthine crystalluria were detected. No bacteria were evident in the urine. Two months later, a UPC of 0.04 (RR < 0.3) was measured. On day 625, a borderline hematocrit (0.37 l/l, RR 0.37–0.63), thrombocytopenia (119 × 10^9^/l, RR 148–484 × 10^9^/l), and hyperglobulinemia (51 g/l, RR 25–45 g/L) were present, comparable to the findings on day 0, except for the raise in hematocrit. On day 679, worsening hyperproteinemia (88 g/L, RR 52–82 g/L), hyperglobulinemia (60 g/l, RR 25–45 g/L), and azotemia, with a creatinine of 23.3 mg/l (RR 5–18 mg/l), were found. However, albumin levels were within normal limits (28 g/l, RR 23–40 g/l, Table 2). IFAT titer stayed at the same level (1:640) compared with day 0. PCR testing for *Leishmania* was performed on blood and bone marrow, and revealed a moderate and high parasite load, respectively. Besides allopurinol, meglumine antimoniate treatment was again initiated (Table 1). After almost 6 weeks on meglumine antimoniate, the dog was reevaluated on day 736 and its clinical parameters were within normal limits, but the blood tests still revealed a mild but improving azotemia with a creatinine level of 19.4 mg/l (RR 5–18 mg/l), a persisting mild anemia (0.35 l/l, RR 0.37–0.63), and improving hyperglobulinemia (53 g/l, RR 25–45 g/L) (Table 2). Quantitative PCR on blood and bone marrow was still positive.

**Fig. 2:**
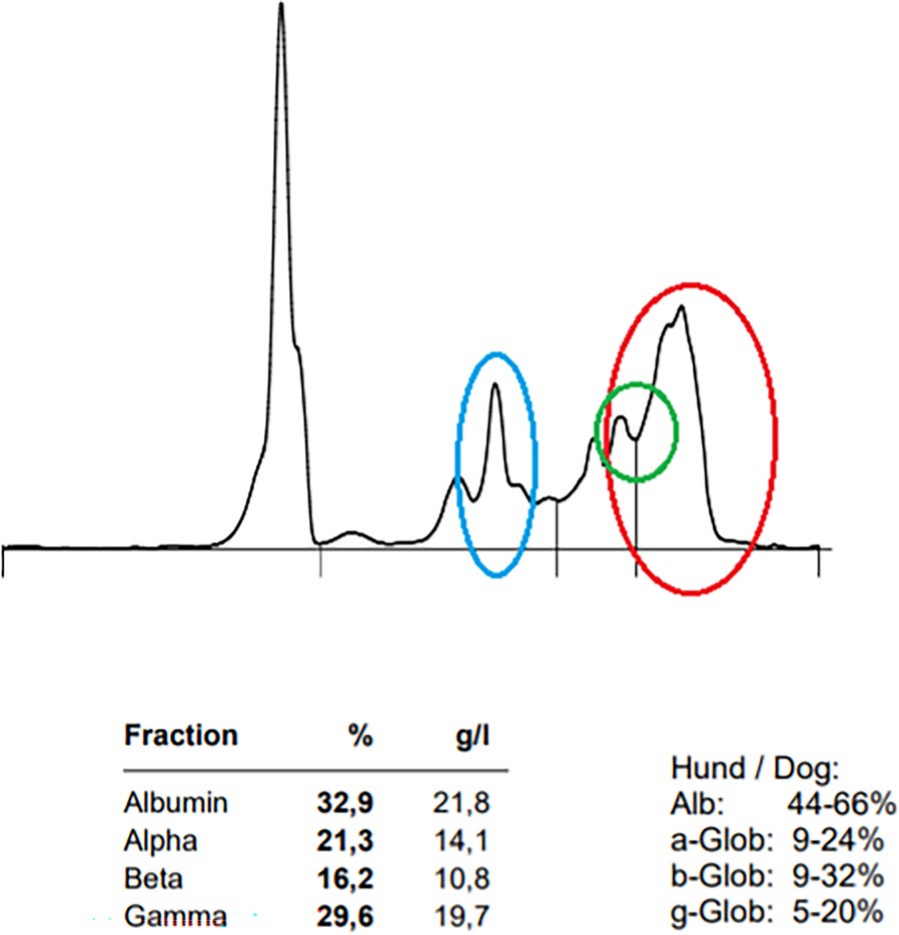
Serum protein capillary electrophoresis in a 2-year-old female, spayed Akita Inu infected with *Leishmania infantum* with polyclonal peaks in the alpha-2 (blue circle) and gamma sections (red circle), and beta–gamma bridging (green circle)

On day 1322, the dog was still in good condition and maintained the same body weight. Physical examination revealed mild splenomegaly and prescapular, as well as popliteal, lymph adenomegaly. Hematology in the LABOKLIN laboratory revealed mild thrombocytopenia (93 × 10^9^/l, RR 150–500 × 10^9^/l), and serum biochemistry showed a mild hyperproteinemia (75.9 g/l, RR 54–75 g/l) with hyperglobulinemia (46.5 g/l, RR < 45 g/l), a marked elevation of the CRP (38.2 mg/l, RR < 15.0 mg/l), and decreased iron concentration (10.4 µmol/l, RR 15–45 µmol/l; Table 2). Serum protein electrophoresis showed an alpha-2 peak, a beta-gamma bridging, and a polyclonal peak in the gamma sections (Fig. 2). The UPC was significantly elevated at 2.5 (RR < 0.2), and a urinary specific gravity of 1.020 (RR 1.016–1.040) was noted in combination with xanthine crystalluria.

*Leishmania* ELISA serology was positive (21.5 LE, < 11 LE negative), as well as the IFAT (1:1024, ≤ 1:64 negative, Table 2). The quantitative PCR blood test was positive, with low concentrations of *Leishmania* (10 *Leishmania*/ml EDTA blood) (Table 2). Testing for resistance against allopurinol revealed a *METK* gene CN of below 3, confirmed by running the drug resistance panel LeishGenR (NANO1HEALTH SL, Bellaterra, Barcelona, Spain) at Nano1Health SL.

## Discussion

This case report is the first to describe a potential correlation between the decrease in the CN of the *Leishmania METK* gene and the clinical findings suggestive of resistance in a dog with a relapse of canine leishmaniasis. The current treatment protocol [15] recommends treatment with pentavalent antimonials, to rapidly reduce parasite load and clinical signs, combined with prolonged allopurinol treatment, which has a mainly parasitostatic action. Studies have shown that the use of allopurinol reduces the frequency of relapses [24, 25]. In the case described herein, treatment with antimonials induced, as expected, a clear clinical improvement, but after months of treatment, clinical relapses occurred, indicating a lack of efficacy of allopurinol.

The use of PCR testing to reveal CNs is a fast, reliable, and cost-effective diagnostic method. Direct PCR testing of tissues represents a great advantage compared with testing resistance by culturing the parasite; this includes isolation of the parasite and growing it in increasing levels of allopurinol, which is a laborious, expensive, and lengthy procedure requiring months of work, and not suitable for providing fast results as needed in clinics.

The reliability of the determination of the CN of the *METK* gene is based on a critical level of *L. infantum* parasites in the sample tested. The quantification in the presented dog revealed a low parasitemia of 10 *Leishmania*/ml EDTA blood. Therefore, testing bone marrow instead of EDTA blood might have provided higher yields of *L. infantum* and therefore higher accuracy in the diagnostic method used for determination of the CN. Invasive bone marrow sampling was declined by the dog owners, although it is a common procedure in clinical practice. However, the clinical relapse of the disease accompanied by hematological and biochemical results, as well as the reported low CN of the *METK* gene, supported a strong suspicion of resistance to allopurinol in the presented dog.

Until recently, limited data regarding cutoff values for reduction in the *METK* gene CN and clinical suspicion of resistance were available. A reduction of the CN was demonstrated in two *Leishmania* isolates with clinical suspicion for resistance and in four isolates with experimentally induced resistant clonal strains, revealing an average relative quantity value of 1.02 ± 0.08, while the average for susceptible ones was 1.48 ± 0.04, with statistically significant differences between both groups (*P* = 0.017) [26]. These numbers are equivalent to two and three copies, which suggest a deletion of one copy of this locus in the resistant clones [26]. Therefore, a cutoff of < 3 CNs can be stated as suspicious for resistance, but further studies and clinical cases suspicious for resistance are needed for confirmation.

The diagnosis of *L. infantum* infection was based on the cytological finding of amastigotes in the spleen, bone marrow, and in the liver; a positive qPCR of blood and bone marrow; as well as a positive serology by IFAT and ELISA. Additionally, hematological and biochemical abnormalities consistent with a *L. infantum* infection were noted (Tables 2 , 3). In our dog, treatment of 1316 days with allopurinol was followed by a clinical relapse of *L. infantum* infection, verified by positive PCR testing of peripheral blood, positive IFAT, and ELISA testing. This diagnosis was accompanied by thrombocytopenia, hyperglobulinemia, elevation of CRP, and a decrease in iron concentration. All mentioned hematological and biochemical abnormalities are consistent with canine leishmaniasis [15, 27]. The *Leishmania* titer increased from 1:640 to 1:1024 following the clinical relapse. However, the increase must be interpreted with care because, owing to the measurement in two different diagnostic laboratories, making comparisons between the titers is challenging [27]. Additionally, the difference can be interpreted as tolerable interassay and interobserver variability. An ELISA titer of 22.1 LE represents a moderate elevation in the LABOKLIN laboratory [27].

**Table 3:** Complete blood count, biochemical parameters, and parasitological status in a 2-year-old female, spayed Akita Inu infected with *Leishmania infantum* in the LABOKLIN laboratory (Bad Kissingen, Germany) on day 1322 after first presentation

Parameter	Reference interval	Values
Complete blood count^a^		
Red blood cells (× 10^12^/l)	5.5–8.5	**8.76**
Hemoglobin (g/l)	150–190	**132**
Hematocrit (l/l)	0.44–0.52	0.47
White blood cells (× 10^12^/l)	6.0–12.0	7.1
Segmented (× 10^9^/l)	3.0–9.0	3.4
Bands (× 10^9^/l)	< 0.6	0.0
Lymphocytes (× 10^9^/l)	1.0–3.6	3.4
Eosinophils (× 10^9^/l)	0.04–0.6	0.1
Monocytes (× 10^9^/l)	0.04–0.5	0.3
Hypochromasia	Negative	Negative
Anisocytosis	Negative	Negative
Platelets (× 10^9^/l)	150–500	**93**
Biochemistry^b^		
Alpha-amylase (U/l)	< 1,650.0	1,618
DGGR-lipase (U/l)	< 120.0	31.8
Glucose (mmol/l)	3.05–6.1	5.2
Fructosamine (µmol/l)	< 374	253
Triglycerides (mmol/l)	< 3.9	0.95
Cholesterol (mmol/l)	3.1–10.1	7.2
Bilirubin (µmol/l)	< 3.4	< 0.1
Alkaline phosphatase (U/l)	< 147	64
Glutamate dehydrogenase (U/l)	< 8.0	2.0
Gamma-glutamyl transferase (U/l)	< 10.0	1.7
Alanine transaminase (U/l)	< 88.0	23.7
Aspartate aminotransferase (U/l)	< 51.0	27.5
Creatine kinase (U/l)	< 200.0	47.0
Total protein (g/l)	54.0–75.0	**75.9**
Albumin (g/l)	25.0–44.0	29.4
Globulin (g/l)	< 45.0	**46.5**
Urea (mmol/l)	3.3–8.3	4.7
Creatinine (µmol/l)	< 125	122
Phosphorus (mmol/l)	0.7–1.6	0.8
Magnesium (mmol/l)	0.6–1.3	0.9
Calcium (mmol/l)	2.3–3.0	2.4
Sodium (mmol/l)	140–155	141
Potassium (mmol/l)	3.5–5.1	4.3
Iron (µmol/l)	15–45	**10.4**
C-reactive protein (mg/l)	< 15.0	**38.2**
*Leishmania* testing		
*Leishmania* IFAT^c^	≤ 1:64	**1:1024**
*Leishmania* ELISA^d^	< 11 LE	**22.1**
*Leishmania* qPCR (/ml EDTA blood)	—	**10 (positive)**

*Segmented* segmented neutrophilic granulocytes, *Bands* banded neutrophilic granulocytes, *EDTA* ethylenediamine tetraacetic acid, *ELISA* enzyme-linked immunosorbent assay, *IFAT* immunofluorescence antibody test, *qPCR* quantitative polymerase chain reaction,

^a^Sysmex XN-V analyzer, Sysmex Deutschland, Norderstedt, Germany

^b^Cobas 8000, Roche Deutschland Holding GmbH, Mannheim, Germany

^c^MegaFLUO^®^ LEISH, MegaCor Diagnostik GmbH, Hörbranz, Austria

^d^NovaTec VetLine Leishmania ELISA, Immundiagnostica GmbH, Dietzenbach, Germany

Bold values are outside the reference intervals of the commercial LABOKLIN laboratory (Bad Kissingen, Germany)

In the monitoring of canine leishmaniasis, special focus should be put on the renal and inflammatory status of the dog infected with *Leishmania* [15, 27]. The UPC is useful for evaluating the risk of chronic kidney disease [28], and proteinuria is a negative prognostic factor in canine leishmaniasis [29]. In our dog, an increase in the UPC was indicative of treatment failure and the suspicion of allopurinol resistance. For monitoring the inflammatory status, the CRP is a very reliable diagnostic tool. Usually, in dogs responding well to treatment, a decrease in CRP can already be noted within a timeframe of 2 weeks after starting antileishmanial therapy, with values in the reference ranges after about 4 weeks [30–32]. Owing to the relatively short half-life of this acute-phase protein, it reacts much quicker compared with serum protein electrophoresis and/or serological titers [27]. Serological titers usually start to decrease in a period of 30 days, if the dog responds well to therapy [33, 34]. However, some individual dogs do not show a decrease in antibodies even in a period of 6 months [35], and some treated infected dogs do not reach antibody titers that are in the reference range even after successful treatment and clinical control of disease [36]. Therefore, qPCR testing for *Leishmania* spp., ideally out of bone marrow, and determination of the *METK* CN are very helpful methods in dogs with suspected treatment failure and/or resistance against allopurinol, as shown in this case report.

Clinical relapse of canine leishmaniasis can be supported by the increase of *Leishmania* concentration revealed by qPCR testing, cytopenia (mono-, bi-, pancytopenia) in hematology, worsening hyperproteinemia with hyperglobulinemia, an increase in CRP, and monitoring of the UPC, as demonstrated in this case report. If PCR testing of bone marrow is not possible, lymph node aspirates and/or EDTA blood can be used. In EDTA blood, the sensitivity can be lower owing to smaller numbers of *Leishmania* amastigotes circulating in the peripheral blood [37].

Relapse of canine leishmaniasis after long-term application of allopurinol, either alone or combined with meglumine antimonate and/or miltefosine, has been described previously [35, 38–41]. However, an association between resistance to antileishmanial drugs and disease relapse with drug resistance substantiated by genetic testing of variation in gene CNs has rarely been documented [17–19], with only one study investigating *L. infantum* isolates from dogs with clinical relapse of leishmaniasis [20]. The reduction in the *METK* gene was found to be typical of resistance to allopurinol in *L. infantum* isolates from dogs with relapse, owing to resistance to this drug, and in *L. infantum* strains that were induced to develop resistance in a previous well-detailed study [26].

Owing to the suspicion of primary IMHA in the beginning, treatment with prednisolone was reduced stepwise and stopped on day 722, most likely not influencing the clinical relapse of leishmaniasis on day 1316, almost 2 years after the end of steroid therapy. Mild to moderate nonregenerative anemia is closely associated with canine leishmaniasis [15] and represents a negative prognostic factor for sick dogs [42]. The nonregenerative anemia found in the dog during its relapse was most likely secondary to leishmaniasis and was not primary immune-mediated, where a regenerative anemia with reticulocytosis is expected. Therefore, the anemia was most likely a consequence of the clinical relapse.

A lack of therapeutic effectiveness of antimonial drugs could not be demonstrated as described above. The relapses were related to the administration of allopurinol as the only prescribed drug for two periods of more than one and a half years, respectively. Owing to the results of the *METK* CN assay, the relapses were most likely associated with a lack of efficacy of allopurinol, and raised the clinical suspicion of resistance to it. Additionally, reinfection with a new *L. infantum* strain that caused the clinical relapse cannot be ruled out completely.

However, in conclusion, the consistency of clinical relapse of canine leishmaniasis, the hematological and biochemical results, the outcomes of *L. infantum* qPCR and serology, and the reduction in the CN of the *METK* gene were interpreted as highly suspicious for resistance against allopurinol in the presented dog. PCR testing is a cost-effective, fast, reliable, objective, and highly specific diagnostic tool for determination of CNs in the *METK* gene in dogs with clinical suspicion of resistance against allopurinol.

Resistance against antileishmanial drugs should be considered as a potential underlying cause in dogs with relapse of canine leishmaniasis, especially if allopurinol is used for long-term management. Dogs with clinical *L. infantum* infections are also highly infectious to sand flies [43], and therefore dogs infected with allopurinol-resistant strains may provide a great risk for infection of naïve dogs, cats, and humans.

## Data Availability

No datasets were generated or analyzed during the current study.

## Abbreviations

CN: Copy number
CRP: C-reactive protein
ddPCR: Digital droplet polymerase chain reaction
DNA: Deoxyribonucleic acid
ELISA: Enzyme-linked immunosorbent assay
EDTA: Ethylenediaminetetraacetic acid
*GAPDH*: Glyceraldehyde-3-phosphate dehydrogenase
IFAT: Indirect fluorescent antibody test
IgG: Immunoglobulin G
IMHA: Primary immune-mediated hemolytic anemia
LE: Laboratory specific units
*METK*: *S*-adenosylmethionine synthetase
PCR: Polymerase chain reaction
qPCR: Quantitative polymerase chain reaction
UPC: Urinary protein-to-creatinine ratio
